# A user exposure based approach for non-structural road network vulnerability analysis

**DOI:** 10.1371/journal.pone.0188790

**Published:** 2017-11-27

**Authors:** Lei Jin, Haizhong Wang, Binglei Xie, Le Yu, Lin Liu

**Affiliations:** 1 School of Architecture and Urban Planning, Harbin Institute of Technology, Shenzhen, Guangdong, P.R. China; 2 School of Civil and Construction Engineering, Oregon State University, Corvallis, OR, United States of America; 3 School of Municipal and Environmental Engineering, Harbin Institute of Technology, Harbin, Heilongjiang, P.R. China; Beihang University, CHINA

## Abstract

Aiming at the dense urban road network vulnerability without structural negative consequences, this paper proposes a novel non-structural road network vulnerability analysis framework. Three aspects of the framework are mainly described: (i) the rationality of non-structural road network vulnerability, (ii) the metrics for negative consequences accounting for variant road conditions, and (iii) the introduction of a new vulnerability index based on user exposure. Based on the proposed methodology, a case study in the Sioux Falls network which was usually threatened by regular heavy snow during wintertime is detailedly discussed. The vulnerability ranking of links of Sioux Falls network with respect to heavy snow scenario is identified. As a result of non-structural consequences accompanied by conceivable degeneration of network, there are significant increases in generalized travel time costs which are measurements for “emotionally hurt” of topological road network.

## Introduction

During the past decades, modern highly social development of urbanization and rapid growth of population have led to more reliance on road network to meet the citizens’ social life demand. Thus, road network vulnerability analysis has long been a key topic in transportation science. Road network vulnerability, which is usually caused by negative incidents, e.g. natural hazards and extreme events, can bring about a considerable reduction in transportation function of road network. An efficient evaluation for road network vulnerability makes much sense to identify the susceptibility to negative incidents which usually weaken the road network performance. That is, road network vulnerability greatly influences the performance of network vehicular flows. Therefore, this paper aims to develop a methodology for travel cost-based road network vulnerability analysis, including both the travel cost metrics for vulnerability after the extreme events’ occurrence and the recognition of vulnerable links which would be a critical component resulting in congestion during a given period. An effective road network vulnerability index is newly proposed by considering travel costs of links without topological degradation in a dense urban road network threatened by external strains.

### Current methodologies

Considering the current articles illustrating the road network vulnerability, two main steps are usually adopted to investigate it: firstly differentiating categories of vulnerability based on the consequences of negative incidents, and secondly proposing an evaluation method to quantify the negative effects. Regarding the differentiating vulnerability, Berdica [1] emphasized that vulnerability analysis for the road transportation system should be studied as a problem of an insufficient level of service. Then regarding the evaluation methods, several metrics have been proposed for investigating road network vulnerability, mainly expressed in three aspects of reliability, accessibility and serviceability. First of all, for the reliability, the leading researcher Bell [2–6] focused on the characterization of transport network based on pure mathematics of graph theory. Then with considerations of accessibility, the leading researchers, Chen [7, 8], Taylor [9, 10] and Jenelius [11, 12], mostly focused on travel cost-based accessibility analysis based on rank of link criticality or area importance. Then considering the serviceability, researchers usually put much interest in investigating the travel cost-based serviceability analysis based on rank of link criticality [13, 14] and evaluation of link capacity [15–18].

In terms of quantifying vulnerability, the existing vulnerability analysis studies for road network have displayed a common view that simulation methods can be applied to research on the links failures impacted by negative incidents and quantify the accompanied negative effects. Among the developed simulation methods, comparing topology changed and unchanged statuses of a road network by simulating physical closures of links is a widely used one. Then the received rank lists of links criticality were regarded as indices of network vulnerability [3, 5, 8, 9, 12, 15]. For the sparse road network of a region-level area, it is suitable to simulate links physical failures to describe negative effects caused by natural hazards and extreme events. For a dense urban road network, it is indeed fact that some critical links would be closed due to natural hazards and extreme events. They are typically more critical to the network functioning, i.e., degenerative accessibility associated with the occurrence of vulnerability incidents. The degeneration of network functioning could be measured by the change of travel cost-based network performance. However, most of the time the road network topology would not change under natural hazards and extreme events. That means physical closures are not definite results derived from negative external strains. Additionally, in a dense urban road network, drivers with restricted route choice sets have no choice but to use a low-functioning link which was always regarded as a failure one in current road vulnerability analysis framework [19–22]. Both non-structural negative consequences and metrics for non-structural dense road network vulnerability from the perspective of travel costs have not been addressed.

### Objective of research

This study raises a novel topic that how to analyze and evaluate the dense urban road network vulnerability without structural negative consequences (i.e., topology disruptions). The methodological framework developed in this study addresses two main objectives. The *first* objective of this study is to develop a methodological framework for investigating the road network vulnerability without structural negative consequences. This is achieved by studying vulnerability as negative consequences of external strains which should be considered as “emotionally hurt” of road network. The *second* objective is to propose a general network vulnerability index for dense urban road network vulnerability in a global network-wide perspective based on travel cost analysis. It bears close connections to the foundations of micro-economic user exposure and non-structural negative consequences, which can bypass the complex task of links spanning procedure.

### Contribution

The central contribution of this paper is to show how user exposure approach, based on variant road conditions, can be used to evaluate the non-structural road network vulnerability. The derivation of new evaluation method is achieved by quantifying generalized travel costs under non-structural negative scenarios. It is shown that evaluation of non-structural road network vulnerability provides transportation controller with a easy way to identify potential vulnerable elements in road networks. Taken as a whole, the proposed methodology and obtained results of this study would contribute to better investigating dense urban road network vulnerability without structural negative consequences.

### Outline of paper

The rest of the paper is organized as follows. Section 2 develops a methodological framework to investigate the non-structural road network vulnerability resulting from external negative incidents. Section 3 uses the proposed framework to measure the road vulnerability of a cold region which is threatened by regular heavy snow incidents. Finally, section 4 concludes the paper.

## Methodology

### Vulnerability analysis: Structural versus non-structural

This section introduces a novel methodological framework of road network vulnerability analysis by considering non-structural negative consequences and travel cost-based user exposure. It addresses a real problem that there are no links physical closures under the influence of negative incidents which would weaken the road networks’ service level.

If we trace the linguistic roots of the English noun *vulnerability*, then we find that it is derived from the word *vulnerable*. The Collins English Dictionary (Online Edition, https://www.collinsdictionary.com/dictionary/english/vulnerable) defines *vulnerable* as, capable of being physically or emotionally wounded or hurt. Specifically, vulnerability is accompanied by two possible facts: physically wounds or emotionally hurt. In the field of road network vulnerability analysis, the widely used definition adopted by Berdica [1] is that vulnerability is the susceptibility to incidents that can result in considerable reductions in road network serviceability. To cope with the reasons of considerable reductions in a road network’s service level, researchers usually paid more attention to the simulations with just considering the role of structural negative consequences, but the role of varying road conditions and its socioeconomic impacts for users are often neglected.

However, structural negative consequences are just half of the reflections of road network vulnerability, like physically wounds to vulnerability. The non-structural negative consequences are another important expressions of the road network vulnerability, like emotionally hurt to vulnerability. In fact, when an negative incident happens, it may not only lead to network disabilities, but also increase the network sensibility to internal and external strains which also greatly affect the road network service. For internal strains, daily incidents such as traffic accidents would reduce road capacity due to the turbulence effects of lane changing behaviors in the context of car-following theory [23, 24]. For external strains, extreme weather conditions such as heavy snow or heavy rain would then decrease the road capacity on account of the slippery road surface. Therefore, bad road conditions and complex traffic conditions would both reduce the level of service of a road network, and the reductions will expand when these conditions become worse [25, 26]. Thus, the network functioning would be degraded due to negative effects without structural disruptions, which can be measured by travel cost-based user exposure.

This paper mainly discusses the method for measuring road network vulnerability with non-structural negative consequences. The non-structural negative consequences are resulted from external strains. The vulnerability of road network is measured by degeneration of vehicular traffic operation of a topological road network [27, 28]. In order to measure vulnerability of dense road network with non-structural negative consequences, the quantification of negative consequences is first proposed. Then the negative effects of non-structural negative consequences are evaluated by travel cost-based user exposure approach.

### Quantifying negative consequences: User equilibrium model accounting for variant road conditions

#### Road capacity accounting for variant road conditions

As a result of external strains, i.e., extreme weather conditions with heavy snow or heavy rain, negative consequences would result in bad road conditions. Generally, compared to the normal condition, the vehicular capacity of road with slippery surface would suffer a significant decline under the external strains. The vehicular capacity of road segment is a concept related to the maximum sustainable flow rate under given geometric and environmental conditions. Accordingly, the maximum sustainable flow rate means that vehicles can be reasonably expected to traverse a uniform segment of roadway during a specified time period. Therefore, the basic vehicular capacity for a uniform road segment of a topological road network can be computed according to a generalized equation as follows,

$$\begin{array}{c} C=\frac{3600}{T_{min}}=\frac{3600}{L_{min}/\left(V/3.6\right)}=\frac{1000V}{L_{min}} \end{array}$$ (1)

where *C* is the basic vehicular capacity, also the number of vehicles per hour; *T*_*min*_ is the minimum safe headway, in seconds (s); *L*_*min*_ is the minimum safe headway, in meters (m); *V* is the certain speed of vehicular flow, in kilometers per hour (km/h).

With the considerations of varying road conditions and negative consequences, the minimum safe headway *L*_*min*_ can be decomposed into four categories, that is vehicle driving distance *L*_1_, vehicle braking distance *L*_2_, length of vehicle body *L*_3_, and safe braking distance *L*_4_ during the driver’s reaction time *t*. The formula of vehicular capacity adopted in this paper is,

$$\begin{array}{c} C=\frac{1000V}{L_{min}}=\frac{1000V}{L_{1}+L_{2}+L_{3}+L_{4}}=\frac{1000V}{\frac{V}{3.6}t+\frac{V^{2}}{254\varphi }+L_{3}+L_{4}} \end{array}$$ (2)

where *φ* is the coefficient of friction between tires and surfaces for roads, which depends on road conditions.

According to Eq (2), the vehicular capacity is a function of the average speed of traffic flow and the coefficient of friction for roads. Based on this function, we can calculate the road capacity as the basis for evaluating road impedance under variant road conditions, such as dry roads in normal weather conditions, frozen roads in heavy snowy days and wet roads in heavy rainy days.

#### User equilibrium model

When vehicles are travelling in a dense urban road network, the travel time costs are not in direct proportion to travel distance. Especially, the traffic volume and road capacity have severe impacts on travel time in bad road conditions. Therefore, travel time is chosen to display road impedance. Consider a road network *G* be a directed graph denoted by a set *N* of nodes and a set *A* of directed links. For a link *a*, the link travel time *t*_*a*_ can be calculated by the Bureau of Public Roads (BPR) function as follows:

$$\begin{array}{c} T_{a}=\frac{L_{a}}{V_{f}}\left[1+\alpha {\left(\frac{q_{a}}{C_{a}}\right)}^{\beta }\right] \end{array}$$ (3)

where *L*_*a*_ is the length of link *a*, and *a* ∈ *A*; *V*_*f*_ is the free-flow speed on link *a*; *q*_*a*_ is the traffic flow of link *a*; *C*_*a*_ is the vehicular capacity of link *a*; *α* and *β* are the delay parameters which are dependent on road conditions.

According to the principle of user equilibrium [29], travelers always choose least-cost paths from origin to destination. When traffic flows reach to the network equilibrium state, we can obtain the link flows and link travel time costs through the Beckmann model [30]. However, if road conditions of links have been changed, travelers would have to re-select their paths again. Therefore, for deterministic network demand **q**, the assignment results can be calculated by using the Beckmann model. Then the obtained results are used to calculate the differences in travel time costs of network between a normal state and a degeneration state. The established model is shown as follows:

$$\begin{array}{c} \min\;\;\sum_{a\in A}\int_{0}^{x_{a}}t_{a}\left(\omega \right)d\omega \end{array}$$ (4)

s.t.

$$\{\begin{array}{l} q_{rs}=\sum_{k\in W_{rs}}f_{k}^{rs},\;\forall r\in R,s\in S;\\ f_{k}^{rs}\geq 0,\;\forall r\in R,s\in S,k\in W_{rs}\\ x_{a}=\sum_{r\in R}\sum_{s\in S}\sum_{k\in W_{rs}}f_{k}^{rs}\cdot \delta_{a,k}^{rs}\;\forall a\in A \end{array};$$ (5)

where *x*_*a*_ is the link traffic flow of link *a*; *t*_*a*_ is the link travel time of link *a*; *t*_*a*_(*x*_*a*_) is the function of link traffic flow *x*_*a*_ of link *a*; $f_{k}^{rs}$ is the path traffic flow of path *k* from origin *r* to destination *s*; $\delta_{a,k}^{rs}$ is a link-path incidence variable, $\delta_{a,k}^{rs}=1$ if path $p_{k}^{rs}$ uses link *a*, otherwise $\delta_{a,k}^{rs}=0;$ *A* is a set of links; *R* is a set of origins; *S* is a set of destinations; *W*_*rs*_ is a set of paths from origin *r* to destination *s*; *q*_*rs*_ is mean travel demand from origin *r* to destination *s*.

Suppose that all travellers try to choose the shortest path in order to minimize their travel time costs. The objective function is strictly convex and leads to a unique solution. In an user equilibrium state, the travel time costs on all used paths between each O-D pair are equal to or less than the travel time costs on any unused paths. Traditional link-based solution algorithms can be used to solve the above model, such as the penalty function method and Lagrange multiplier method. Thus, the solutions of the above user equilibrium model, link traffic flow *x*_*a*_ and link travel time cost *t*_*a*_, can be obtained to measure the performance of road network. According to the principle of user equilibrium, the cost of any traveller from origin *r* to destination *s* is equal to the minimum path travel time cost *u*_*rs*_, which can be calculated by

$$\begin{array}{c} u_{rs}=\sum_{a\in A}\sum_{k\in W_{rs}}t_{a}\cdot \delta_{a,k}^{rs}. \end{array}$$ (6)

### Evaluating negative effects: The new non-structural vulnerability index with the perspective of user exposure

Some metrics for road network vulnerability have been proposed to evaluate the negative consequences of links closures resulted from negative incidents. Among them, the generalized travel costs metrics [8, 11–14, 31], accessibility metrics [9, 10], link criticality and area importance metrics [11, 12, 31, 32], network efficiency metrics [27], or some changes in topology parameters [3, 5, 6] are well-known ones. However, they were usually used to simulate the negative effects of links closures caused by external negative incidents, such as natural hazards, extreme events. Instead, evaluations on non-structural road network vulnerability have not yet been discussed in the existing vulnerability literature.

#### User exposure

In this study, a new evaluation index from the perspective of *user exposure* is proposed to measure non-structural road network vulnerability. It aims to quantify the degeneration of vehicular traffic operation of a topological road network, mainly concentrating on the negative effects on users caused by external strains. Firstly, the concept of user exposure [11, 12, 31] was adopted to measure the change in generalized cost of a single user under a certain disruption scenario of network structure as

$$\begin{array}{c} Ex\left(n|\sigma \right)=\Delta TC\left(n|\sigma \right)=TC\left(n|\sigma \right)-TC\left(n|\sigma_{0}\right) \end{array}$$ (7)

where *Ex*(*n*|*σ*) is the exposure of user *n* under a certain disruption scenario *σ* of network structure; Δ*TC*(*n*|*σ*) is the change in generalized travel cost of user *n* under a disruption scenario *σ* of network structure; *TC*(*n*|*σ*_0_) is the generalized travel cost of user *n* under a normal scenario *σ*_0_ before the occurrence of scenario *σ*; *TC*(*n*|*σ*) is the generalized travel cost of user *n* under a disruption scenario *σ*.

#### The new non-structural vulnerability index

In the context of non-structural road network vulnerability analysis, the degeneration of vehicular traffic operation of the topological road network is typically measured by the difference between normal network and degraded network impacted by non-structural negative consequences. Therefore, the metric for user exposure under disruption scenario is extended to non-structural degeneration scenario resulted from external strains. For a road network *G*, exposure of user *n* to non-structural degeneration scenario *μ* can be calculated by

$$\begin{array}{c} Ex\left(n|\mu \right)=\Delta TC\left(n|\mu \right)=TC\left(n|\mu \right)-TC\left(n|\mu_{0}\right)=u_{rs}\left(n|\mu \right)-u_{rs}\left(n|\mu_{0}\right) \end{array}$$ (8)

where *Ex*(*n*|*μ*) is the exposure of user *n* under a certain non-structural degeneration scenario *μ*; Δ*TC*(*n*|*μ*) is the change in generalized travel cost of user *n* under scenario *μ*; *TC*(*n*|*μ*_0_) is the generalized travel cost of user *n* under the normal scenario *μ*_0_ before the occurrence of scenario *μ*; *TC*(*n*|*μ*) is the generalized travel cost of user *n* under scenario *μ*; *u*_*rs*_(*n*|*μ*_0_) is the minimum path travel time of user *n* under scenario *μ*_0_; *u*_*rs*_(*n*|*μ*) is the minimum path travel time of user *n* under scenario *μ*.

Besides the exposure of user *n* under a certain non-structural degeneration scenario *μ*, a problem that should be addressed efficiently is to compare and rank the vulnerability of links based on the metrics of user exposure. Exposure of user *n* to degeneration of link *a* under a certain non-structural degeneration scenario *μ* can be calculated by

$$\begin{array}{c} Ex\left(n|\mu ,a\right)=\Delta TC\left(n|\mu ,a\right)=TC\left(n|\mu ,a\right)-TC\left(n|\mu_{0},a\right)=t\left(n|\mu ,a\right)-t\left(n|\mu_{0},a\right) \end{array}$$ (9)

where *Ex*(*n*|*μ*, *a*) is the exposure of user *n* to degeneration of link *a* under a certain non-structural degeneration scenario *μ*; Δ*TC*(*n*|*μ*, *a*) is the change in generalized travel cost of user *n* to degeneration of link *a* under scenario *μ*; *TC*(*n*|*μ*_0_, *a*) is the generalized travel cost of user *n* to degeneration of link *a* under scenario *μ*_0_; *TC*(*n*|*μ*, *a*) is the generalized travel cost of user *n* to degeneration of link *a* under scenario *μ*; *t*(*n*|*μ*_0_, *a*) is the link travel time of user *n* to degeneration of link *a* under scenario *μ*_0_; *t*(*n*|*μ*, *a*) is the link travel time of user *n* to degeneration of link *a* under a non-structural degeneration scenario *μ*.

Based on the above postulations, aggregating user exposure measures is mainly considered to simplify the ranking of vulnerability of links, and used as a tool for assessing the performances of road network or its components. Thus, the change in travel cost by exposure of users, who use link *a* as follows, can be measured.

$$\begin{array}{c} \begin{array}{cc} \Delta Ex(\mu ,a) & =Ex\left(\mu ,a\right)-Ex\left(\mu_{0},a\right)=\sum_{n\in g}TC\left(n|\mu ,a\right)-\sum_{n\in g_{0}}TC\left(n|\mu_{0},a\right)\\ & =\sum_{n\in g}t\left(n|\mu ,a\right)-\sum_{n\in g_{0}}t\left(n|\mu_{0},a\right)=x_{a}^{\mu }\cdot t\left(n|\mu ,a\right)-x_{a}^{\mu_{0}}\cdot t\left(n|\mu_{0},a\right) \end{array} \end{array}$$ (10)

where Δ*Ex*(*μ*,*a*) is the change in travel cost of link *a*; *Ex*(*μ*_0_,*a*) is the travel cost of link *a* under a normal scenario *μ*_0_; *Ex*(*μ*,*a*) is the travel cost of link *a* under a non-structural degeneration scenario *μ*; *g* is an aggregate group of users who use link *a*, and *g* = *n*_1_,⋯,*n*_*x*_*a*__; *x*_*a*_ is the link traffic flow solved by Eq (4).

The vulnerability of urban road transportation system can be evaluated in terms of non-structural negative consequences. In this paper, the non-structural negative consequences are represented by the relative change in travel cost of link *a* between a non-structural degeneration scenario *μ* and a normal scenario *μ*_0_. Thus, the vulnerability of dense road network *VUL*(*μ*,*a*) can be formulated as follows:

$$\begin{array}{c} VUL\left(\mu ,a\right)=\frac{\Delta Ex(\mu ,a)}{Ex(\mu_{0},a)}=\frac{Ex\left(\mu ,a\right)-Ex\left(\mu_{0},a\right)}{Ex(\mu_{0},a)}. \end{array}$$ (11)

In conclusion, based on the above framework of vulnerability analysis, the ranking of vulnerability of links can be measured by the exposure of user group *g* to the degeneration of link *a* under a certain non-structural degeneration scenario *μ* of network *G*.

## Application

This section mainly displays the applications of the proposed methodological framework of non-structural road network vulnerability analysis. A field vulnerability analysis of the medium-size road network of Sioux Falls was conducted as a case study. The non-structural negative consequences resulted from heavy snowfall are detailedly discussed. Three steps are covered: introducing the network structure and the weather of Sioux Falls, quantifying the non-structural negative consequences resulted from heavy snow, identifying the vulnerability of road links of the network.

### Road network geometry and local climate of Sioux Falls

**Fig 1:**
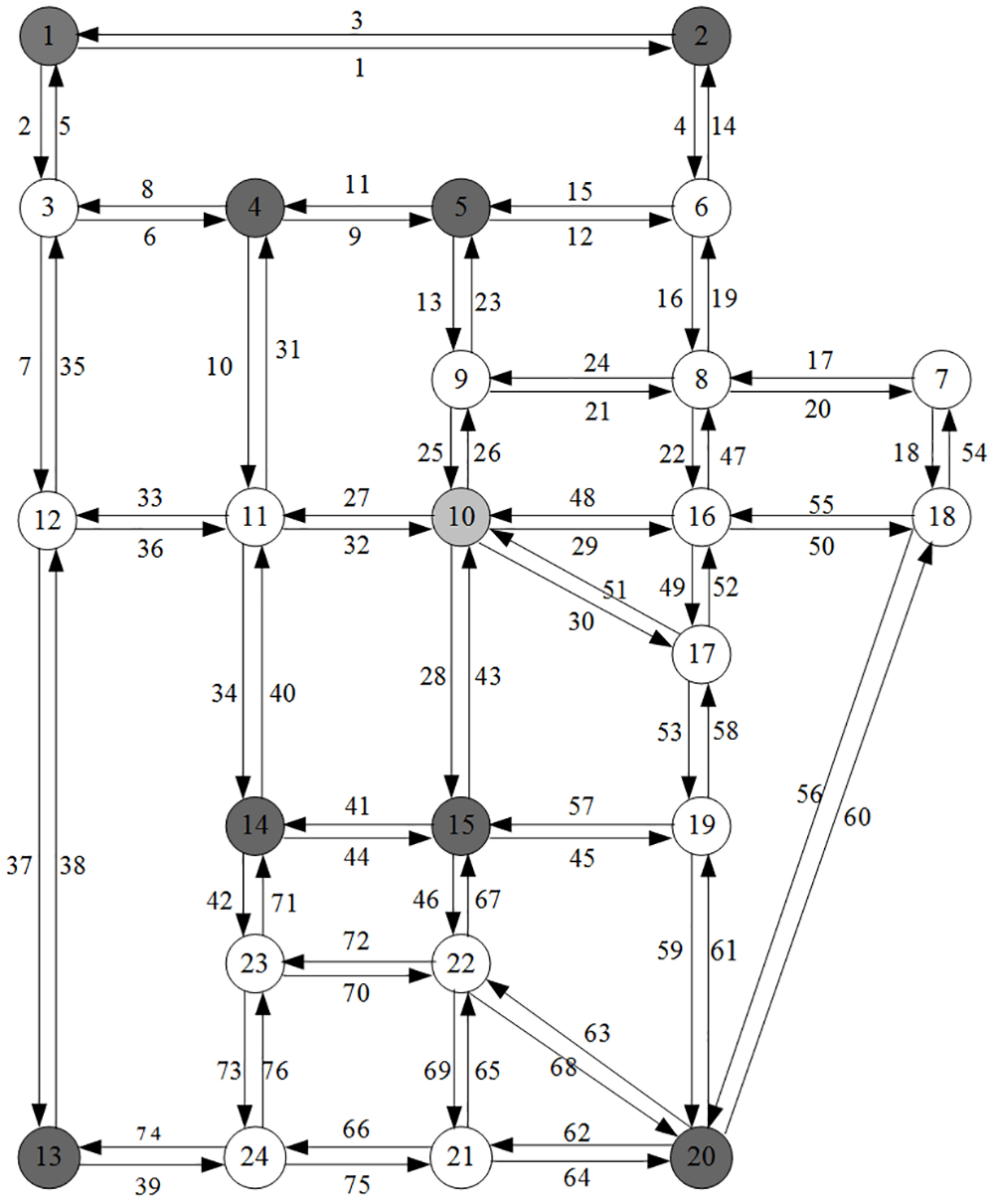
Sioux Falls road network.

Sioux Falls is a major metropolitan area in the southeastern part of South Dakota. The Sioux Falls road network shown in Fig 1 consists of 24 nodes and 76 links. In this numerical experiments, it is assumed that there are 8 traffic analysis zones and 56 O-D pairs. The network characteristics of the selected numerical objects, such as the O-D demands and link parameters, were mainly cited from Kongsomsaksakul’s dissertation [33]. The local climate of Sioux Falls is characterized by long-term cold winters. During the wintertime, the average temperatures vary from -13.9°C to -0.4°C, and snowfalls mostly occurred in light or moderate amounts varying from 17.8 cm to 21.1 cm on average snowy days and totaling 113 cm during the whole winter season. The weather characteristics in Sioux Falls area derived from the website http://w2.weather.gov/climate/xmacis.php?wfo=fsd. Further detailed climatic data for Sioux Falls area are shown in Table 1.

**Table 1:** Climate data for Sioux Falls, South Dakota (1981–2010).

Month	Jan	Feb	Mar	Apr	Oct	Nov	Dec	Year
Average high (°C)	−3.1	−0.4	6.2	14.7	15.3	5.8	−1.9	13.7
Average low (°C)	−13.9	−11.3	−5.2	1.3	2.3	−5.2	−12.3	1.6
Record low (°C)	−39.0	−41.0	−31.0	−16.0	−21.0	−27.0	−35.0	−41.0
Snowfall in depth (cm)	20.1	17.8	21.1	11.4	3.3	18.8	20.8	113.3
Snowy days (≥ 0.25 cm)	7.2	6.2	5.3	2.4	0.8	4.1	7.0	32.9

### Non-structural vulnerability analysis of the Sioux Falls network

Generally, a winter storm (heavy snow) has been denoted by the National Weather Service of the United States that snowfalls amounts are usually between 10 cm to 18 cm or more. Then as shown in Table 1, a total of six months present higher average snowfall amounts between 11.4 cm and 21.1 cm, which can be regarded as “heavy snow” according to the criteria mentioned above. Under such heavily snowy weather conditions, road capacity and relative parameters should be clearly determined to specify the corresponding road networks of Sioux Falls for non-structural vulnerability analysis. Thus, the parameters for the vehicular capacity formula in Eq (2) and BPR function in Eq (3), were set as below. The coefficient of friction *φ* was set at 0.15 for snow roads. The length of vehicle body *L*_3_ was set at 5m for all vehicles. The safe braking distance *L*_4_ during the driver’s reaction time was set at 32m. The delay parameters *α* and *β* were set as 0.75 and 0.26, respectively. The traffic free-flow speed *V*_*f*_ of snowy road was set as 55km/h.

As a result of snow and ice events, there are usually severe consequences caused by external strains from the heavy snowfall in cold regions. For simplicity, the vehicular capacity of roadway with slippery surface is used to quantify the negative consequences resulted from heavy snow in a cold region. It would reduce greatly than normal scenario. Then The procedure proposed to quantify non-structural negative consequences resulted from heavy snow was applied to calculate the user equilibrium model. Equilibrium solutions of the above model are then used to calculate the values of user exposure and vulnerability.

Table 2 shows a ranking list of non-structural vulnerability index for Sioux Falls road network under heavy snow conditions. 76 links were checked for their vulnerability and degeneration under a heavy snow scenario. It can be noted that the most vulnerability value is 1.711 at link 33, the weighted generalized travel time cost of which increased by 171.1%. The least vulnerability value is 0.417 at link 51, and its weighted generalized travel time cost increased by 41.7%. The vulnerability values of links ranged from 0.417 to 1.711, with an overall average value 1.239. It shows that the weighted generalized travel time cost increased by 123.9% under a heavy snow scenario.

**Table 2:** Rank and values of non-structural vulnerability index for Sioux Falls road network under heavy snow condition.

Rank	Link ID	Vulnerability value	Rank	Link ID	Vulnerability value
1	33	1.711	39	41	1.237
2	36	1.689	40	56	1.233
3	19	1.667	41	60	1.233
4	4	1.632	42	17	1.217
5	14	1.632	43	75	1.217
6	29	1.605	44	70	1.200
8	40	1.600	45	61	1.200
7	16	1.600	46	49	1.200
9	48	1.579	47	53	1.200
10	31	1.556	48	73	1.200
11	10	1.556	49	68	1.184
12	34	1.467	50	38	1.174
13	74	1.433	51	37	1.174
14	39	1.433	52	8	1.167
15	32	1.421	53	57	1.133
16	15	1.400	54	45	1.133
17	47	1.395	55	59	1.133
18	27	1.395	56	65	1.133
19	71	1.367	57	69	1.133
20	25	1.348	58	72	1.133
21	26	1.348	59	6	1.133
22	13	1.342	60	44	1.132
23	23	1.342	61	55	1.130
24	7	1.333	62	63	1.105
25	42	1.333	63	64	1.089
26	35	1.333	64	50	1.087
27	28	1.311	65	11	1.067
28	43	1.311	66	18	1.067
29	66	1.304	67	54	1.067
30	20	1.304	68	67	1.033
31	2	1.300	69	46	1.033
32	5	1.300	70	3	1.000
33	12	1.300	71	1	0.956
34	22	1.289	72	9	0.933
35	52	1.267	73	21	0.427
36	58	1.267	74	24	0.427
37	76	1.267	75	30	0.417
38	62	1.267	76	51	0.417

**Fig 2:**
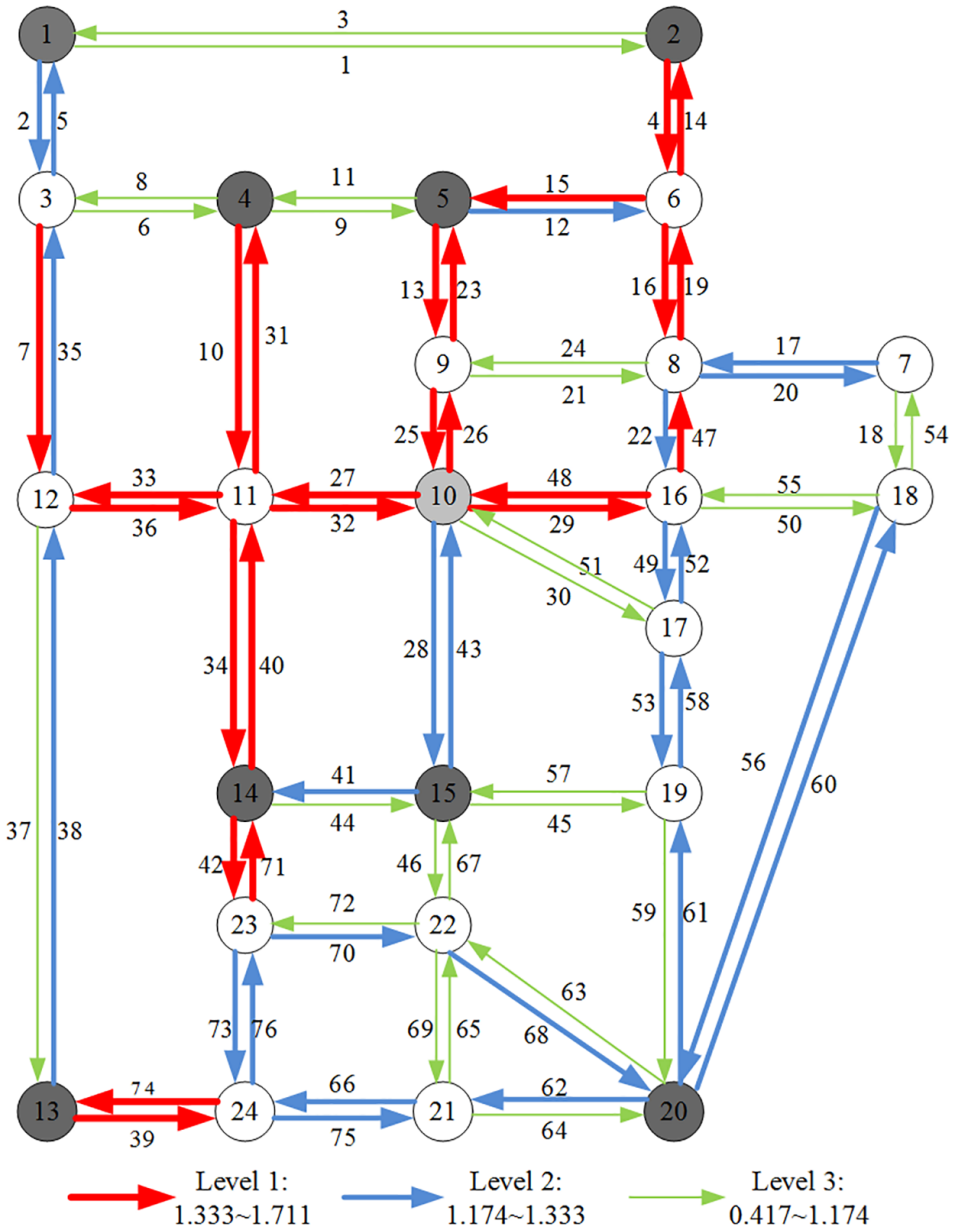
Non-structural vulnerability of Sioux Falls road network under heavy snow condition.

As a result of network degeneration due to a winter storm event, a set of critical links have been evaluated by the change of user exposure under a winter storm scenario. Fig 2 presents the non-structural vulnerability index with respect to user exposure. Observed from the vulnerability links, interestingly, the links in the central part of road network have turned out to be critical (e.g. links originated at Node 11). The most critical links are the Link 33 and Link 36 originated at Node 11. The high level travel demands of the central part of Sioux Falls network may lead to travel costs increase. Equally, the results is also able to identify network partitions as it considers the vulnerability of substantial links. It is clear that different traffic zones experience different degrees of vulnerability.

## Conclusions

This paper has proposed a non-structural road network vulnerability analysis framework with the consideration of generalized travel time costs increases resulted from external negative consequences. Aiming at some parts of network which become sensitive to internal and external strains without structural disruptions, the proposed methodological framework makes it possible to evaluate the considerable reduction of network function. Based on the rationale of micro-economic approach, i.e., link performance function and UE model, a new vulnerability index regarding user exposure was proposed to measure the negative consequences of variant road conditions resulted from external negative incidents at the network-wide level.

Then a case study was conducted by using this methodological framework to investigate the vulnerability of a dense road network threatened by regular heavy snow incidents. Ranking results of vulnerability showed that variant road conditions influenced by heavy snow have significant impacts on generalized travel costs, which helps to pick out flow bottlenecks in the field of congestion prediction. In addition, the ideas can also be extended to evaluate other non-structural road network vulnerability resulted from both internal and external strains, such as vulnerability measured by network degeneration caused by turbulence effects of traffic accidents and vulnerability resulted from heavy rain on rainy days. In particular, by considering the results of non-structural road network vulnerability analysis under extreme weather conditions, governments could better issue weather warnings for adjusting transportation actions of road network users.

## Supporting information

S1 Fig The Sioux Falls road network. (TIF)

S2 Fig Non-structural vulnerability of Sioux Falls road network under heavy snow condition. (TIF)

S1 Table Available traffic data used in this study. (XLSX)
