# Supplementary figures and images for: A user exposure based approach for non-structural road network vulnerability analysis
Source: PLoS One. 2017 Nov 27;12(11):e0188790. doi: 10.1371/journal.pone.0188790 (PMC5703479; doi:10.1371/journal.pone.0188790)

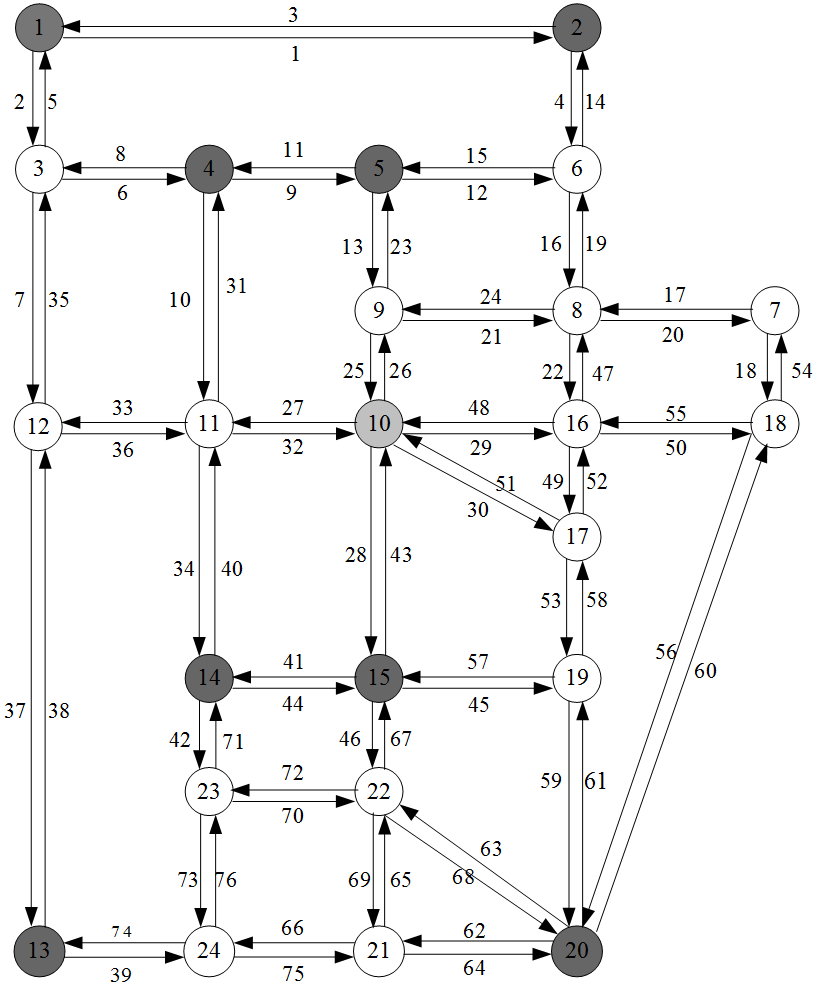

Supplement: S1 Fig — (TIF) [file pone.0188790.s001.tif]

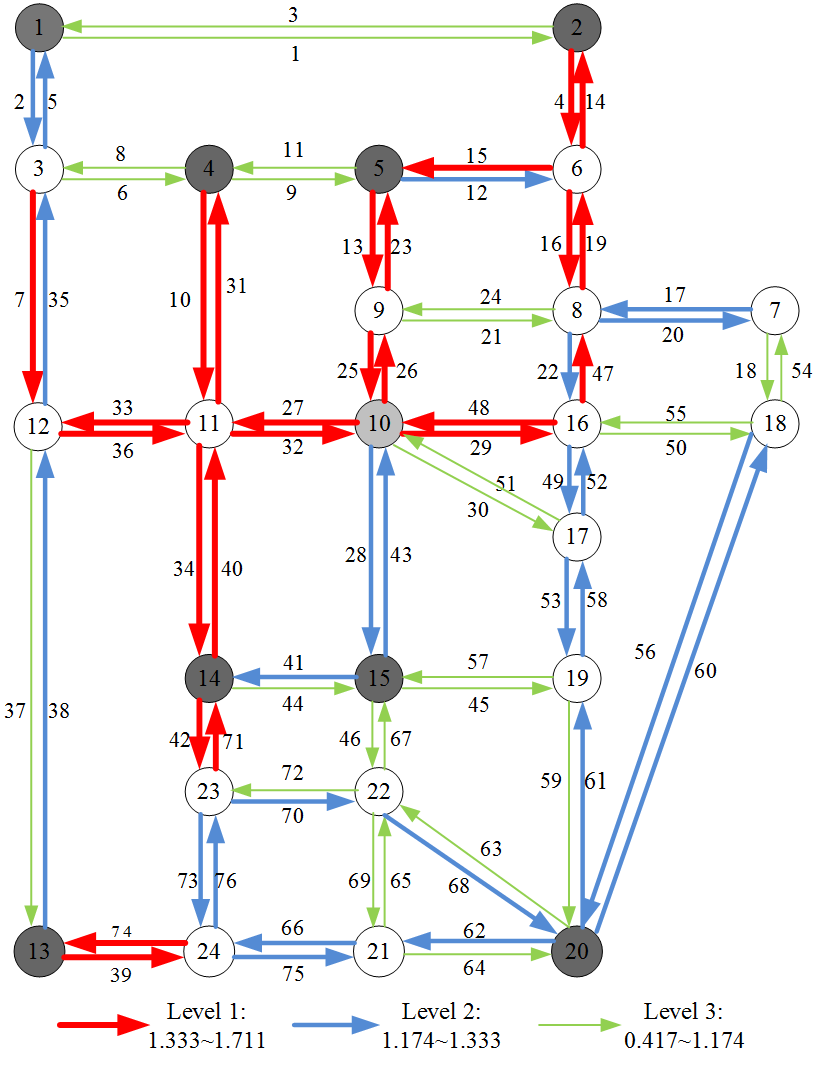

Supplement: S2 Fig — (TIF) [file pone.0188790.s002.tif]
